# Ibrutinib: A New Frontier in the Treatment of Chronic Lymphocytic Leukemia by Bruton’s Tyrosine Kinase Inhibition

**DOI:** 10.2174/1871525712666140115143914

**Published:** 2013-12

**Authors:** Ajoy Lawrence Dias, Dharamvir Jain

**Affiliations:** 1 Division of Hematology and Medical Oncology, James Graham Brown Cancer Center, University of Louisville Health Sciences Center, Louisville, Kentucky, USA;; 2 Division of Hematology and Medical Oncology, James Graham Brown Cancer Center, University of Louisville Health Sciences Center, Kentucky, USA

**Keywords:** B cell receptor (BCR), Bruton’s tyrosine kinase (BTK), Chronic lymphocytic leukemia (CLL), Ibrutinib.

## Abstract

Chronic lymphocytic leukemia (CLL) is characterized by progressive accumulation of nonfunctional mature B cells in blood, bone marrow and lymphoid tissues. In the last decade, our understanding of CLL and consequently our diagnostic and therapeutic approaches have changed dramatically. Conventional fludarabine based chemotherapy has led to improved disease response and longer survival in young patients with CLL. However its application in elderly patients has been restricted by substantial myelosuppression and infection. Treatment of CLL is now moving towards targeted therapy. The success of new class of agents such as monoclonal antibodies, proteasome inhibitors and immunomodulatory derivatives has sparked further search for treatment agents with novel targets to inhibit. The B cell receptor activating pathway involving the Bruton’s tyrosine kinase (BTK) is crucial in B cell production and maintenance and is an attractive therapeutic target. Ibrutinib is an oral covalent inhibitor of the BTK pathway that induces apoptosis of B cells. Early phase studies with Ibrutinib either as a single agent or in combination regimens have shown promising results with an excellent safety profile in patients with high-risk, refractory or relapsed CLL and elderly treatment-naïve patients. This review summarizes the current knowledge of Ibrutinib in the treatment of CLL.

## INTRODUCTION

Chronic lymphocytic leukemia (CLL) is a post germinal center neoplasm characterized by clonal proliferation and accumulation of mature appearing lymphocytes in the blood, bone marrow, lymph nodes and spleen. The CLL cells are typically B cell in origin. It is estimated that 15,680 people in the United States will be diagnosed with and 4,580 will die of CLL in 2013. With an age-adjusted incidence of 4.3/100,000 men and women per year in the United States, CLL is the most common type of leukemia in western countries [1]. From 2006 to 2010, the median age at diagnosis for CLL was 71, and the median age at death was 79 years, making this a disease predominantly affecting the older people [1]. Most patients are asymptomatic and diagnosis is frequently made on a routine blood count. The leukemic cells usually co-express CD5 and CD23, and the diagnosis can be established by the demonstration of ≥5x10^9^/L monoclonal cells with this phenotype in the peripheral blood, even in the absence of lymphadenopathy, organomegaly or other clinical features [2]. Current therapies are effective in inducing remission in most patients who can tolerate them but these therapies are not curative, and resistance ultimately develops [3]. In addition, many older patients are not candidates for the newer chemoimmuno-therapy regimens like fludarabine, cyclophosphamide and rituximab (FCR) due to their underlying comorbidities and therapy related toxicities. Therefore, alternative approaches that remain effective in relapsed disease and which are well tolerated become the need of the hour [4]. Early clinical experiences with targeted kinase inhibitors suggest that these drugs have the potential to do both and have therefore generated a great excitement in the CLL community [5]. 

## BRUTON’S TYROSINE KINASE (BTK) PATHWAY

Bruton’s tyrosine kinase (BTK) is a member of the Tec family of kinases. It is thought to be a cytoplasmic protein, expressed predominantly on the B lymphocytes, lymphocyte precursors and developing myeloid cells, but is absent in the plasma cells and T cells [6]. The central role of BTK in B cell function is underscored by the human disease X-linked agammaglobulinemia, which is caused by the loss of function mutations in BTK. Loss of function of the BTK gene inhibits B lymphocytes production due to a maturation inhibition between the pro- and pre-B cell stages. This inhibition causes an inability to make all classes of immunoglobulins, leading to recurrent severe bacterial infections and increased susceptibility to viral and parasitic infections. In addition, these mutations result in virtual absence of B cells [7, 8]. 

## B CELL RECEPTOR (BCR)

Among the various signals from the microenvironment, B cell receptor (BCR) activation and signaling, particularly in lymphatic tissues, has emerged as a key mechanism. However the precise mechanism of BCR stimulation and the nature of the antigen(s) that activate the BCR remain largely unknown. BCR activation sets in motion an orderly cascade of signaling events that ultimately promotes B cell selection, proliferation, differentiation, apoptosis and cell migration, which are essential for the functioning and survival of both normal and malignant B cells [9]. Besides CLL, BCR signaling also has been identified as a central pathologic mechanism in diffuse large B cell lymphoma, mantle cell lymphoma and follicular lymphoma [9, 10].

## B CELL SIGNALING

**Fig. (1):**
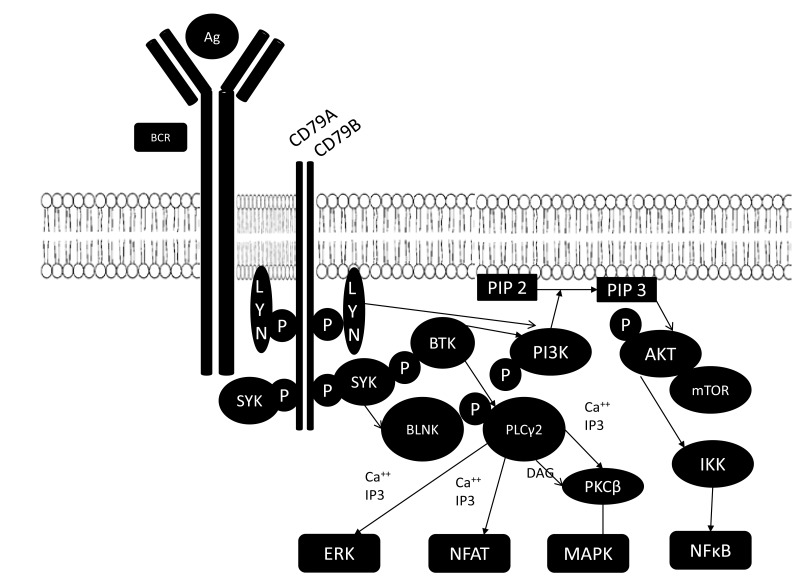
The BCR pathway. Abbreviations: AKT, Activated protein kinase B; BCR, B cell receptor; BLNK, B cell linker protein; BTK, Burton’s tyrosine kinase; DAG, diacylglycerol; ERK, extracellular regulated kinase; IKK, inhibitor of κB kinase; MAPK, mitogen activated protein kinase; mTOR, mammalian target of rapamycin; NFAT, nuclear factor of activated T- cells; NFκB, nuclear factor κB; PI3K, phosphatidyl inositol-3 kinase; PLCγ2, Phospholipase Cγ2; PKC, protein kinase C; SYK, spleen tyrosine kinase.

The BCR is composed of an antigen specific membrane immunoglobulin (Ig) paired with Ig-alpha/Ig-beta hetero-dimers (CD79a/CD79b) (Fig. **1**). BCR activation leads to Phosphatidyl Inositol 3 Kinase (PI3K) activation with generation of phosphatidylinositol-3, 4, 5-triphosphate (PIP3). After sufficient accumulation of PIP3, BTK is recruited at the inner surface of the plasma membrane. Initial experiments demonstrated that BTK then undergoes phosphorylation at site Y551 by the Src family kinases Blk, Lyn and Fyn [11]. This then leads to autophosphorylation at Y223. Phosphorylated BTK then activates phospholipase Cγ2 (PLCγ2), which leads to activation of downstream effectors protein kinases Cβ (PKCβ), and ultimately the transcription factor, nuclear factor-kappa B (NF-kB) [12]. These events result in increased proliferation and survival of B cells, mediated by upregulation of transcription factors, mainly NF-kB. Although the most prominent role of NF-kB is to inhibit apoptosis, it also induces transcription of the BTK gene [13]. Stimulation of this pathway has been shown to aid in the proliferation and prolonged survival of B cells in lymphoid malignancies [14].

Although there is plethora of evidence demonstrating the vital nature of BTK for B cell proliferation and differentiation, there is also data which demonstrates that BTK plays a role in apoptosis. It has been identified as the first dual function regulator of apoptosis [15]. Further studies have shown that BTK mediated apoptosis is dependent on membrane localization and kinase activity and involves p38 MAPK [16].

## IBRUTINIB (PCI-32765) - AN ORAL BTK INHIBITOR

**Fig. (2):**
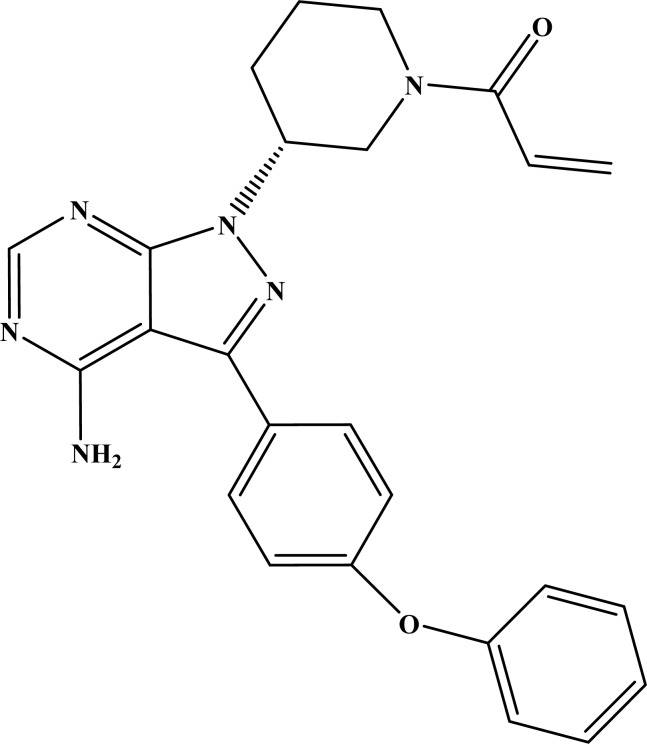
Structure of Ibrutinib (1-[3R)-3-[4-amino-3-(4-phenoxyphenyl) pyrazolo [3, 4-d] pyrimidin-1-yl] piperidin-1-yl] prop-2-en-1-one).

Ibrutinib (1-[(3*R*)-3-[4-amino-3-(4-phenoxyphenyl) pyrazolo [3, 4-d] pyrimidin-1-yl] piperidin-1-yl] prop-2-en-1-one) is an orally available irreversible potent inhibitor of BTK (Fig. **2**). 

It binds covalently to the cysteine-481 amino acid of the BTK, resulting in inhibition of kinase activity with IC_50_ of 0.5 nM [17]. Ibrutinib does have significant activity (IC_50 _<100nM) against 10 other kinases, including seven with a cognate cysteine residue. These include BLK, BMX, ITK, TEC, EGFR, ERBB2, and JAK3 [18]. In the B cell lymphoma cell line DOHH2, a fluorescently tagged derivative of Ibrutinib bound only to BTK, was able to block autophosphorylation of PLCγ and ERK in response to BCR pathway activation. A concentration of 10 nM was sufficient to fully occupy the active site of BTK and the blockade was irreversible [18]. With this sufficiently encouraging data, the efficacy of the drug was tested in spontaneous lymphomas in dogs, and three of eight dogs showed partial response [18].Multiple groups have now studied the effects of Ibrutinib in CLL cells *in vitro*. Ibrutinib has modest activity on apoptosis *in vitro*, but it blocks signaling and activation in response to BCR and CD40 pathway stimulation and disrupts the protective effect of stromal cell co-incubation [19]. Ibrutinib can also block integrin mediated adhesion to fibronectin as well as well as signaling and homing in response to CXCL12 and CXCL13 [20, 21]. Secretion of cytokines CCL3 and CCL4, normally stimulated by BCR activation, is also markedly reduced by Ibrutinib [21]. Taken together, these data suggest that Ibrutinib may work at least in part by modulating the interaction between CLL cells and the microenvironment, rather than by direct cytotoxicity alone. 

Ibrutinib induced inhibition of BCR, NF-kB, and ERK signaling occurs very quickly as demonstrated in the lymph nodes and is sustained in the bone marrow during treatment. The strong and sustained reduction in proliferation and activation of CLL cells in the tissue microenvironment suggests that BTK is indeed a central hub mediating the nourishing and protective effects of the tumor microenvironment^1^.

## EARLY CLINICAL STUDIES

Clinical studies of Ibrutinib in CLL started in 2010. The initial phase I study enrolled 56 patients with B cell lymphomas including CLL with a median of three prior regimens. They were evaluated in five cohorts with punctuated dosing (28 days on, 7 days off) and two cohorts with continuous dosing [22]. The mean plasma concentrations were obtained 1-2 hours after drug administration. Mean half-life was 4-8 hours with no evidence of drug accumulation after repeated dosing. No dose limiting toxicities were observed and the drug was well tolerated. BTK occupancy was assessed by a competitive binding assay in which the probe binds to a target cysteine residue in the absence of the drug, but is excluded from binding when the drug has previously bound. Using this assay, BTK occupancy was found to be complete at doses ≥ 2.5 mg/kg. Although the half-life of the drug is 6-11 hours, occupancy and BTK inhibition remain complete for 24 hours due to the irreversible covalent nature of binding. Based on these data, doses of 420 and 840 mg were selected for future studies. In this phase I study, the overall response rate across all histologies was 60%, with a 79% overall response rate in the 14 CLL patients [22]. A 75% response rate in four mantle cell lymphoma patients was also notable. 13 follicular lymphoma patients had a relatively poor response rate of 23%, while six diffuse large B cell lymphoma patients had a 17% response rate. Most adverse effects were grade 1 or 2, the most common being diarrhea, fatigue and cough. Very few patients developed grade 3 or 4 hematological toxicities, which included anemia, thrombocytopenia and all these were dose dependent^2^ [22]. CLL was therefore considered a disease of significant interest for further study. Also of interest in this study was the observation that CLL patients started on the drug had rapid shrinkage of lymph nodes along with an increase in lymphocyte count that would revert back to baseline on the punctuated schedule when the drug was stopped.

## IBRUTINIB IN RELAPSED/REFRACTORY CLL

Encouraging results from the phase I studies prompted a phase Ib/2 multicenter study to assess the efficacy, safety, pharmacokinetics and pharmacodynamics of Ibrutinib in relapsed or refractory CLL or small lymphocytic lymphoma [23]. A total of 85 patients, the majority of whom were considered to have high risk disease, were enrolled in the study. Two cohorts of patients (27 patients in cohort 1 and 24 patients in cohort 3) were assigned to receive a fixed daily dose of 420 mg of Ibrutinib. Cohort 2 comprised of 34 patients, and was assigned to receive a continuous daily dose of 840 mg. The median age of the cohort was 66 years and had received a median of four previous therapies. The high risk features included 57% patients with bulky lymphadenopathy with lymph node diameters greater than 5 cm ( with 13% having ≥10 cm lymphadenopathy), and 41% were fludarabine refractory. 72% of patients had at-least one poor risk molecular feature which included del 17p13.1 in 33%, del 11q22.3 in 36%, unmutated *IgVH* in 69%. At a median follow up of 20.9 months, 54 patients (64%) were still receiving treatment and 31(36%) had discontinued treatment due to various reasons. The overall response rate, according to standard International Workshop on CLL 2008 criteria (IWCLL 2008), was 71% (2 complete responses and 34 partial responses) in the 420 mg cohort and 71% in the 840 mg cohort. In addition, 10 patients in the 420 mg cohort (20%) and 5 patients in the 840 mg cohort (15%) had a partial response with persistent lymphocytosis. Blood lymphocytosis was generally noted by day 7 (in 78% of the patients); it peaked at a median of 4 weeks and then slowly declined. In 50 of the 63 patients (79%) the lymphocyte count normalized or was reduced by 50% from the baseline level. This increase in lymphocyte count was not considered disease progression in the absence of B symptoms or new cytopenias. Lymphocytosis occurred concomitantly with a notable reduction in lymph node size and spleen size as well as frequent improvement in cytopenias. The response to Ibrutinib did not appear to vary according to the traditional high risk prognostic features, such as 17 p13.1 deletion. The only factor associated with a response was the mutation status of the *IgVH.* Notably, 4 of the 12 patients with mutated* IgVH* (33%) had a partial response or complete response and 5 (42%) had a partial response with lymphocytosis. By contrast, 53 of the 69 patients with an unmutated *IgVH* (77%) had a partial response or complete response and 9 (13%) had a partial response with lymphocytosis. At 26 months, the estimated progression free survival rate was 75% and overall survival was 83%. Toxic effects were predominantly grade 1 or 2 and included transient diarrhea, fatigue and upper respiratory tract infection; thus, patients could receive extended treatment with minimal hematologic toxic effects [23].

## IBRUTINIB IN TREATMENT NAÏVE CLL

Thirty one treatment naïve CLL patients older than 65 years were enrolled in phase Ib/II trial, of which twenty six received Ibrutinib 420 mg daily and five received 840 mg daily. The median age of this cohort was 71 years, 43% had non-mutated *IgVH*, with two patients with 17p13.1 deletion. The median follow up duration was 14.4 months for the 420 mg cohort and 7.4 months for the 840 mg cohort. The drug was well tolerated with most frequent side effects being diarrhea, nausea, fatigue and rash. Grade 3-4 toxicities included 13% diarrhea, 10% infection and 12% hematologic toxicity, which included anemia and thrombocytopenia. The overall response rate by IWCLL criteria was 74% with 10% complete response and 61% partial response and an additional 13% had nodal response with lymphocytosis. All but five patients still remain on the study with treatment discontinuation due to adverse events in four patients and progressive disease in one case. The estimated 15 month progression free survival in this treatment naïve group is as high as 95%^3^,^4^.

## IBRUTINIB IN HIGH-RISK CLL

Twenty four patients were grouped as high risk cohort in the parent multicohort phase Ib/II study^4^. The median age was 68 years with a median of four prior treatments. High-risk features included 17 p13.1 deletion in 30% and non-mutated *IgVH* in 83% of patients. They received 420 mg daily dose of Ibrutinib. The median follow up was 10.3 months. The overall response rate by IWCLL criteria was 50% (all partial responses); with 29% achieving partial response with lymphocytosis and 4% of patients progressed while on treatment. The adverse effects were similar to the previous groups, which consisted of diarrhea, fatigue, upper respiratory tract infections, rash, nausea and joint pains^4^.

In another phase II, single center study, Ibrutinib was used as a single agent in the treatment of CLL patients with del 17p13.1, regardless of their prior treatment history. This study enrolled a total of 53 patients, of whom 29 had del 17p13.1; fifteen of the del 17p13.1 patients and eight without del 17p13.1 were treatment naive. At six months, 47 patients were evaluable. Of the patients with del 17p13.1, 53% achieved a partial response and 43% achieved a partial response with lymphocytosis, compared to 82% partial response and 9% partial response with lymphocytosis among the patients without 17p13.1 deletion. The apparent difference in response is due to slower clearance of the treatment-induced lymphocytosis in the del 17p13.1 patients; however, the clinical benefit and disease control in all tissue sites was equal for both cohorts of patients. Twenty-month progression free survival was 100% in the normal 17p13.1 cohort and 85% in the del 17p13.1 cohort. The most common adverse events were predominantly grade 1 and included diarrhea, arthralgia, rash, fatigue, bruising, and cramps. The most common grade 3 or higher adverse events were lung infection (5%) and rash (2%)^5^.

## IBRUTINIB IN COMBINATION THERAPY

The excellent single agent activity of Ibrutinib in refractory and treatment naïve CLL led investigators to test its efficacy when used in combinations with monoclonal antibodies and chemoimmunotherapy to raise the possibility of cure.

### Ibrutinib-Monoclonal Antibody Combinations

Ibrutinib has been studied in combination with rituximab, a genetically engineered chimeric human monoclonal antibody directed against CD20 antigen. Rituximab has been widely used as monotherapy and in combination with chemotherapy in CLL. In a phase 2 study, forty high-risk patients were included to receive a daily dose of 420 mg Ibrutinib in combination with rituximab 375mg/m^2^ weekly for an initial four weeks, followed by daily Ibrutinib with monthly rituximab until cycle 6, and subsequently Ibrutinib alone until disease progression. The high risk features of this cohort included treated or untreated CLL patients with 17p13.1 deletion/TP53 mutation, relapsed CLL with 11q22.3 deletion, and /or progression free survival less than 36 months after chemoimmunotherapy. 17p13.1 deletion or presence of TP53 was observed in 19 of the 40 patients, non-mutated *IgVH* was seen in 31 of the 40 patients, and 11q22.3 deletion was present in 13 patients. 38 patients were evaluable at four month follow up without disease progression. The overall response rate was 83%, with an additional three cases of partial response and persistent lymphocytosis. The treatment was well tolerated with mostly grade 1 to 2 adverse events and only 13 cases of grade 3 or 4 toxicity, which includes neutropenia, fatigue and bone pain which were transient. Biomarker analysis showed a rapid reduction and normalization of plasma levels of the chemokines CCL3 and CCL4, which are secreted in a BCR-dependent manner^6^.

Ibrutinib has been studied in combination with ofatumumab in patients with relapsed/refractory CLL. Ofatumumab is a fully humanized anti-CD20 monoclonal antibody that is approved for the treatment of CLL refractory to fludarabine. As a single agent, it has a response rate of 45% [24]. The phase Ib/II study of Ibrutinib with ofatumumab evaluated three different combination dosing regimens. Twenty seven patients, including three with Ritcher’s transformation, were enrolled in this study with a median age of 66 years and with a median of three prior regimens. Of these, 48% had advanced Rai stage 3-4 disease, 41% were refractory to purine analogues, 91% had unmutated *IgVH*, 37% had 17p13.1 deletion and 33% had 11q22.3 deletion. Patients received Ibrutinib 420 mg daily on a 28 day cycle until disease progression. Ofatumumab was added on day 1 of the second cycle at a dose of 300 mg and then 2000 mg on days 8, 15 and 22 of cycle 2 and days 1, 8 and 15 of cycle 3 and on day 1 only in cycles four to eight. After a median follow up of 6.5 months, the overall response rate was 100% (one complete response) in the CLL/SLL group, and two of the three patients with Ritcher’s transformation achieved partial remission. At a median of 9.8 months, 89% of patients remain on the study, with one off for progressive disease, one to undergo stem cell transplant and one deceased. The most common side effects were similar to the single agent group and were grade 1-2 in severity. The only exception was the neuropathy seen in 25% patients and was more likely related to ofatumumab than Ibrutinib. Ecchymosis was also seen in almost half the patients and likely reflects the inhibition of platelet function by Ibrutinib through a direct effect on BTK^7^. The treatment was therefore well tolerated and highly active in patients with refractory CLL/SLL.

### Ibrutinib with Chemoimmunotherapy

A phase Ib/II study explored the addition of Ibrutinib to bendamustine and rituximab (BR) and with fludarabine, cyclophosphamide and rituximab (FCR) regimen in relapsed refractory CLL patients. The study enrolled 30 patients who had received 1 to 3 prior regimens. For the FCR arm, patients were required to be fludarabine naïve. The FCR arm was closed after enrollment of only three patients due to difficulty in accruing fludarabine naïve patients. In the BR arm, the median age of the cohort was 62 years, with a median of two prior therapies. Patient characteristics and risk factors included bulky disease (53%), Rai stage 3/4 (47%), presence of 17p13.1 deletion (23%), and 11q22.3 deletion (43%). 37% were purine analog refractory and 13% were bendamustine refractory. The treatment program consisted of Ibrutinib 420mg every day, in combination with bendamustine 70mg/m^2^ on days 1-2 and rituximab 375mg/m^2^ in cycle 1, escalating to 500 mg/m^2^ in cycles 2-6. FCR was given at the standard MD Anderson doses to only three eligible and enrolled patients, together with Ibrutinib 420mg per day. The therapy was well tolerated with a median of six cycles of BR and 97% of the planned Ibrutinib dose delivered. 23% of patients required a dose reduction of bendamustine. Toxicity was manageable and included diarrhea, nausea, fatigue and skin rash. Grade 3 hematologic toxicity was present in 17% of patients (grade 4 in 10%) and consisted mainly of neutropenia. Two patients developed tumor lysis syndrome and two developed grade 3 cellulitis. No discontinuations for adverse events were reported. Ninety percent of patients remained in the study. The addition of BR obviated the lymphocytosis and led to a 93% overall response rate, with 13% complete response at a median follow up of 8.1 months. The complete response rate may increase with a longer follow up and upon confirmation with a bone marrow biopsy. The estimated 11 month progression free survival is 90%^8^. These are comparable to the previous study for BR regimen alone, in which the response rate was 59% [25]. Of the three patients in the FCR group, two had bulky disease and none had 17p13.1 or 11q22.3 deletion. The median age was 56 years. All three had been treated with rituximab and lenalidomide on a clinical trial and therefore met the eligibility criteria to be purine analog naïve. FCR-Ibrutinib therapy was well tolerated, all three patients received full six cycles, with one patient requiring dose reduction. The overall response rate was 100% ,with two confirmed absence of minimal residual disease^9^.

The table below summarizes the results of recent trials of Ibrutinib as a single agent and in combination with monoclonal antibodies and chemoimmunotherapy (Table **1**).

## ONGOING TRIALS 

Several phase 3 trials are currently underway, testing efficacy of Ibrutinib with or without bendamustine and rituximab (RESONATE) and Ibrutinib versus Ofatumumab (HELIOS) in relapsed or refractory CLL. RESONATE has already started enrollment. RESONATE 2 will compare Ibrutinib versus chlorambucil in the upfront setting in patients for whom chemotherapy is otherwise contraindicated. The outcome from these phase 3 trials will likely pave the way for US Food and Drug Administration approval of Ibrutinib in management of CLL in appropriate settings.

Several novel BTK inhibitors such as GDC-0824, RN-486, CGI-560, CGI-1746, HM-71224, CC-292, ONO-4059, CNX-774, and LFM-A13 are under active preclinical and clinical development for CLL, B cell NHL and autoimmune disorders such as rheumatoid arthritis and systemic lupus erythematosus. Among these, LFM-A13 represents a first in class dual BTK-PLK inhibitor. These novel inhibitors will likely provide new treatment options not only for B cell lymphomas, but also for autoimmune disorders [26].

## CONCLUSION

Ibrutinib is an oral irreversible inhibitor of BTK which has shown exciting results in the treatment of CLL, likely through modulation of the interaction between the CLL cells and microenvironment as well as through direct cytotoxicity. Although conventional fludarabine based chemoimmuno-therapy has been the standard of care in young and fit patients with CLL, it still carries a risk of serious myelosuppression and infections in the frail and elderly, thereby restricting its use in this group of patients. Ibrutinib now offers an additional therapeutic option in the elderly, difficult to treat patients given its overall superior toxicity profile. In addition, Ibrutinib has shown good response rates in high risk patients especially those with 17p13.1 deletion and unmutated *IgVH,* where conventional chemotherapy has met with poor response and frequent relapses. So far, limited follow-up results have been excellent with regards to progression free survival. However, long term follow up will be needed to demonstrate durable and sustained response with Ibrutinib. It can also be considered as a potential maintenance therapy for high risk patients who have responded to conventional treatment. Further investigations may be undertaken to assess the role of Ibrutinib in the setting of stem cell transplantation either as maintenance therapy or as part of induction treatment. It appears to be an exciting drug with a potential to add a significant therapeutic option in CLL.

1Herman, E.;Mustafa, R.; B S.; Martyr, S.E.; Gyamfi, J.; Valdez, J.; Soto, S.; Farooqui, M.Effective Inhibition Of Tumor Microenvironment Interactions In CLL Patients Treated With The BTK Inhibitor Ibrutinib Results In Sustained Inhibition Of Tumor Proliferation and Survival Pathways.* ASH Annual Meeting Abstracts, *2013, 118.
2Advani, R.; Sharman, J.; Smith, S.; Boyd, T. E.; Grant, B.; Kolibaba, K. S.; Furman, R.; Buggy, J.; Loury, D.; Hedrick, E.; Izumi, R.; Hamdy, A.; Fowler, N. H. The Btk inhibitor PCI-32765 is highly active and well tolerated in patients with relapsed /refractory B cell malignancies: final results from a phase 1 study. *Ann Oncol, *2011, *22*(11 supp 4), 135.
3Byrd, J. C.; Furman, R.; Courte, S. The Bruton's tyrosine kinase (BTK) inhibitor PCI-32765 (P) in treatment naive (TN) chronic lymphocytic leukemia (CLL) patients (pts):interim results of a phase Ib/II study. *ASCO Meeting Abstracts., *2012, *30*(15 supp), 6507.
4Byrd, J. C.; Furman, R. R.; Coutre, S.; Flinn, I. W.; Burger, J. A.; Blum, K. A.; Sharman, J.; Grant, B.; Jones, J. A.; Wierda, W. G.; Zhao, W.; Heerema, N. A.; Johnson, A. J.; Tran, A.; Clow, F.; Kunkel, L.; James, D. F.; O'Brien, S. The Bruton's Tyrosine Kinase (BTK) Inhibitor Ibrutinib (PCI-32765) Promotes High Response Rate, Durable Remissions, and Is Tolerable in Treatment Naive (TN) and Relapsed or Refractory (RR) Chronic Lymphocytic Leukemia (CLL) or Small Lymphocytic Lymphoma (SLL) Patients Including Patients with High-Risk (HR) Disease: New and Updated Results of 116 Patients in a Phase Ib/II Study. *ASH Annual Meeting Abstracts, *2012, *120*(21), 189.
5Farooqui, M.; Aue, G.; Valdez, J.; Martyr, S.; Jones, J.; Soto, S.; Stetler-Stevenson, M.; Yuan, C.; Arthur, D.; Thomas, F.; Tian, X.; Liu, D.; Maric, I.; Wiestner, A. Single Agent Ibrutinib (PCI-32765) Achieves Equally Good and Durable Responses In Chronic Lymphocytic Leukemia (CLL) Patients With and Without Deletion 17p.* ASH Annual Meeting Abstracts, *2013, 673.
6Burger, J. A.; Keating, M. J.; Wierda, W. G.; Hoellenriegel, J.; Ferrajoli, A.; Faderl, S.; Lerner, S.; Zacharian, G.; Huang, X.; James, D. F.; Buggy, J. J.; Kantarjian, H. M.; O'Brien, S. M. The Btk Inhibitor Ibrutinib (PCI-32765) in Combination with Rituximab Is Well Tolerated and Displays Profound Activity in High-Risk Chronic Lymphocytic Leukemia (CLL) Patients. *ASH Annual Meeting Abstracts, *2012, *120*(21), 187.
7Jaglowski, S. M.; Jones, J. A.; Flynn, J. M.; Andritsos, L. A.; Maddocks, K. J.; Blum, K. A.; Grever, M. R.; Geyer, S. M.; Woyach, J. A.; Johnson, A. J.; Heerema, N. A.; Molnar, E.; Stefanos, M.; Devlin, S.; Navarro, T.; James, D. F.; Lowe, A. M.; Hedrick, E.; Byrd, J. C. A phase Ib/II study evaluating activity and tolerability of Btk inhibitor PCI-32765 and ofatumumab in patients with chronic lymphocytic leukemia/small lymphocytic lymphoma (CLL/SLL) and related diseases. *ASCO Meeting Abstracts, *2012, *30*(15_suppl), 6508.
8O'Brien, S. M.; Barrientos, J. C.; Flinn, I. W.; Barr, P. M.; Burger, J. A.; Navarro, T.; James, D. F.; Hedrick, E.; Friedberg, J. W.; Brown, J. R. Combination of the Bruton's tyrosine kinase (BTK) inhibitor PCI-32765 with bendamustine (B)/rituximab (R) (BR) in patients (pts) with relapsed/refractory (R/R) chronic lymphocytic leukemia (CLL): Interim results of a phase Ib/II study. *ASCO Meeting Abstracts, *2012, *30*(15_suppl), 6515.
9Brown, J.; Barrientos, J.; Flinn, I.; Barr, P.; Burger, J.; Navarro, T.; James, D.; Hedrick, E.; Friedberg, J.; O'Brien, S. In *The bruton's tyrosine kinase (BTK) inhibitor Ibrutinib combined with bendamustine and rituximab is active and tolerable in patients with relapsed/refractory CLL, interim results of a phase IB/II study*, EHA Meeting Abstracts, Amsterdam, Haematologica Amsterdam, 2012; p 218.


## Figures and Tables

**Table 1.** Clinical trials with Ibrutinib in CLL.

Study	N	ORR%	CR%	PFS
Single Agent:				
Relapsed/Refractory [23]	85	71	4	75 % at 26 months
Treatment Naive3	31	74	10	96 % at 15 months
High Risk4	24	50	0	NR
High Risk5	29	53	NR	85% at 20 months
Combinations:				
Ibrutinib + Ofatumumab6	27	100	4	89 % on study at 10 months
Ibrutinib + FCR7	3	100	67	100 % at 11 months
Ibrutinib +Rituximab8	40	83	3	NR
Ibrutinib + BR9	30	93	13	90 % at 11 months

N- Number of patients, CLL- Chronic lymphocytic leukemia, ORR-Overall response rate, CR- Complete response, PFS- Progression free survival, BR- Bendamustine and rituximab, NR- Not reported

## References

[1] Howlader N, Noone AM, Krapcho M, Garshell J, Neyman N, Altekruse SF, Kosary CL, Yu M, Ruhl J, Tatalovich Z, Cho H, Mariotto A, Lewis DR, Chen HS, Feuer EJ, Cronin KA (2013). SEER Cancer Statistics Review, 1975-2010.. http://seer.cancer.gov/csr/1975_2010/ Accessed October 25.

[2] Hallek M, Cheson BD, Catovsky D, Caligaris-Cappio F (2008). Guidelines for the diagnosis and treatment of Chronic Lymphocytic Leukemia: a report from the International workshop on Chronic Lymphocytic Leukemia updating the National Cancer Institute - Working Group 1996 Guidelines.. Blood.

[3] Byrd JC, Rai K, Peterson BL, Appelbaum FR, Morrison VA, Kolitz JE, Shepherd L, Hines JD, Schiffer CA, Larson RA (2005). Addition of rituximab to fludarabine may prolong progression-free survival and overall survival in patients with previously untreated chronic lymphocytic leukemia: an updated retrospective comparative analysis of CALGB 9712 and CALGB 9011.. Blood.

[4] Keating MJ, O'Brien S, Albitar M, Lerner S, Plunkett W, Giles F, Andreeff M, Cortes J, Faderl S, Thomas D, Koller C, Wierda W, Detry MA, Lynn A, Kantarjian H (2005). Early results of a chemoimmunotherapy regimen of fludarabine, cyclophosphamide, and rituximab as initial therapy for chronic lymphocytic leukemia.. J. Clin. Oncol.

[5] Davids MS, Brown JR (2012). Targeting the B cell receptor pathway in chronic lymphocytic leukemia.. Leuk. Lymphoma.

[6] Smith CI, Baskin B, Humire-Greiff P, Zhou JN, Olsson PG, Maniar HS, Kjellen P, Lambris JD, Christensson B, Hammarstrom L (1994). Expression of Bruton's agammaglobulinemia tyrosine kinase gene, BTK, is selectively down-regulated in T lymphocytes and plasma cells.. J. Immunol.

[7] Plebani A, Soresina A, Rondelli R, Amato G M, Azzari C, Cardinale F, Cazzola G, Consolini R, De Mattia D, Dell'Erba G, Duse M, Fiorini M, Martino S, Martire B, Masi M, Monafo V, Moschese V, Notarangelo L D, Orlandi P, Panei P, Pession A, Pietrogrande MC, Pignata C, Quinti I, Ragno V, Rossi P, Sciotto A, Stabile A (2002). Clinical, immunological, and molecular analysis in a large cohort of patients with X-linked agammaglobulinemia: an Italian multicenter study.. Clin. Immunol.

[8] Conley ME, Dobbs AK, Farmer DM, Kilic S, Paris K, Grigoriadou S, Coustan-Smith E, Howard V, Campana D (2009). Primary B cell immunodeficiencies: comparisons and contrasts.. Annu. Rev. Immunol.

[9] Stevenson FK, Caligaris-Cappio F (2004). Chronic lymphocytic leukemia: Revelations from the B cell receptor.. Blood.

[10] Chavez JC, Sahakian E, Pinilla-Ibarz J (2013). Ibrutinib: an evidence-based review of its potential in the treatment of advanced chronic lymphocytic leukemia.. Core Evid.

[11] Rawlings DJ, Scharenberg AM, Park H, Wahl MI, Lin S, Kato RM, Fluckiger AC, Witte ON, Kinet JP (1996). Activation of BTK by a phosphorylation mechanism initiated by SRC family kinases.. Science.

[12] Humphries LA, Dangelmaier C, Sommer K, Kipp K, Kato RM, Griffith N, Bakman I, Turk CW, Daniel JL, Rawlings DJ (2004). Tec kinases mediate sustained calcium influx via site-specific tyrosine phosphorylation of the phospholipase Cgamma Src homology 2-Src homology 3 linker.. J. Biol. Chem.

[13] Yu L, Mohamed AJ, Simonson OE, Vargas L, Blomberg KE, Bjorkstrand B, Arteaga HJ, Nore BF, Smith CI (2008). Proteasome-dependent autoregulation of Bruton tyrosine kinase (Btk) promoter via NF-kappaB.. Blood.

[14] Satterthwaite AB, Witte ON (2000). The role of Bruton's tyrosine kinase in B cell development and function: a genetic perspective.. Immunol. Rev.

[15] Uckun FM (1998). Bruton's tyrosine kinase (BTK) as a dual-function regulator of apoptosis.. Biochem. Pharmacol.

[16] Islam TC, Smith CI (2000). The cellular phenotype conditions Btk for cell survival or apoptosis signaling.. Immunol. Rev.

[17] Brown JR (2013). Ibrutinib (PCI-32765): the first BTK (Bruton's tyrosine kinase) inhibitor in clinical trials.. Curr. Hematol. Malig. Rep.

[18] Honigberg LA, Smith AM, Sirisawad M, Verner E, Loury D, Chang B, Li S, Pan Z, Thamm D H, Miller RA, Buggy JJ (2010). The Bruton tyrosine kinase inhibitor PCI-32765 blocks B cell activation and is efficacious in models of autoimmune disease and B cell malignancy.. Proc. Natl. Acad. Sci. USA.

[19] Herman SE, Gordon AL, Hertlein E, Ramanunni A, Zhang X, Jaglowski S, Flynn J, Jones J, Blum KA, Buggy JJ, Hamdy A, Johnson AJ, Byrd JC (2011). Bruton tyrosine kinase represents a promising therapeutic target for treatment of chronic lymphocytic leukemia and is effectively targeted by PCI-32765.. Blood.

[20] de Rooij MF, Kuil A, Geest CR, Eldering E, Chang BY, Buggy JJ, Pals ST, Spaargaren M (2012). The clinically active BTK inhibitor PCI-32765 targets B cell receptor- and chemokine-controlled adhesion and migration in chronic lymphocytic leukemia.. Blood.

[21] Ponader S, Chen SS, Buggy JJ, Balakrishnan K, Gandhi V, Wierda WG, Keating MJ, O'Brien S, Chiorazzi N, Burger JA (2012). The Bruton tyrosine kinase inhibitor PCI-32765 thwarts chronic lymphocytic leukemia cell survival and tissue homing in vitro and in vivo.. Blood.

[22] Advani RH, Buggy JJ, Sharman JP, Smith SM, Boyd TE, Grant B, Kolibaba KS, Furman RR, Rodriguez S, Chang BY, Sukbuntherng J, Izumi R, Hamdy A, Hedrick E, Fowler NH (2013). Bruton tyrosine kinase inhibitor Ibrutinib (PCI-32765) has significant activity in patients with relapsed/refractory B cell malignancies.. J. Clin. Oncol.

[23] Byrd JC, Furman RR, Coutre SE, Flinn IW, Burger JA, Blum KA, Grant B, Sharman JP, Coleman M, Wierda WG, Jones JA, Zhao W, Heerema NA, Johnson AJ, Sukbuntherng J, Chang BY, Clow F, Hedrick E, Buggy JJ, James DF, O'Brien S (2013). Targeting BTK with Ibrutinib in relapsed chronic lymphocytic leukemia.. N. Engl. J. Med.

[24] Coiffier B, Lepretre S, Pedersen LM, Gadeberg O, Fredriksen H, van Oers MH, Wooldridge J, Kloczko J, Holowiecki J, Hellmann A, Walewski J, Flensburg M, Petersen J, Robak T (2008). Safety and efficacy of ofatumumab, a fully human monoclonal anti-CD20 antibody, in patients with relapsed or refractory B cell chronic lymphocytic leukemia: a phase 1-2 study.. Blood.

[25] Fischer K, Cramer P, Busch R, Stilgenbauer S, Bahlo J, Schweighofer C D, Bottcher S, Staib P, Kiehl M, Eckart MJ, Kranz G, Goede V, Elter T, Buhler A, Winkler D, Kneba M, Dohner H, Eichhorst BF, Hallek M, Wendtner CM (2011). Bendamustine combined with rituximab in patients with relapsed and/or refractory chronic lymphocytic leukemia: a multicenter phase II trial of the German Chronic Lymphocytic Leukemia Study Group.. J. Clin. Oncol.

[26] Akinleye A, Chen Y, Mukhi N, Song Y, Liu D (2013). Ibrutinib and Novel inhibitors in clinical development.. J. Hematol. Oncol.

